# Multicenter Study Protocol: Research on Evaluation and Detection of Surgical Wound Complications with AI-Based Recognition (REDSCAR-Trial)

**DOI:** 10.3390/jcm14072210

**Published:** 2025-03-24

**Authors:** Andrea Craus-Miguel, Alejandro Fernández-Moreno, Ana Isabel Pablo-Leis, Marta Romero-Hernández, Marc Munar, Gabriel Moyà-Alcover, Manuel González-Hidalgo, Juan José Segura-Sampedro

**Affiliations:** 1 General and Digestive Surgery Department, Clínica Universidad de Navarra, 31008 Madrid, Spain; 2 PhD Program in Translational Research in Public Health and High Prevalence Diseases, Health Sciences, University of the Balearic Islands (UIB), 07122 Palma, Spain; 3 Outpatient Service, General and Digestive Surgery Nursing Consultation, Hospital Universitario de Fuenlabrada, 28942 Madrid, Spain; 4 Soft Computing, Image Processing and Aggregation (SCOPIA) Research Group, University of the Balearic Islands (UIB), 07122 Palma, Spain; 5 Artificial Intelligence Research Institute of the Balearic Islands (IAIB), University of the Balearic Islands (UIB), 07122 Palma, Spain; 6 Health Research Institute of the Balearic Islands (IdISBa), 07120 Palma, Spain; 7 Computer Graphics and Vision and AI (UGiVIA) Research Group, University of the Balearic Islands (UIB), 07122 Palma, Spain; 8 Laboratory of Artificial Intelligence Applications (LAIA@UIB), University of the Balearic Islands (UIB), 07122 Palma, Spain; 9 General & Digestive Surgery Service, Hospital Universitario La Paz, 28046 Madrid, Spain; juanjose.segura@salud.madrid.org; 10 Hospital La Paz Institute for Health Research (IdiPAZ), 28046 Madrid, Spain; 11 School of Medicine, San Pablo CEU University, 28003 Madrid, Spain; 12 School of Medicine, Alfonso X El Sabio University, 28691 Madrid, Spain

**Keywords:** surgical wound monitoring, smartphone application, artificial intelligence, telemedicine, surgical site infection (SSI), remote monitoring, digital health, postoperative care, telematic follow-up, patient satisfaction, active patient

## Abstract

**Background:** The increasing use of telemedicine in surgical care has shown promise in improving patient outcomes and optimizing healthcare resources. Surgical site infections (SSIs) are a major cause of healthcare-associated infections (HAIs), leading to significant economic and health burdens. A pilot study already demonstrated that RedScar© achieved 100% sensitivity and 83.13% specificity in detecting SSIs. Patients reported high satisfaction regarding comfort, cost-effectiveness, and reduced absenteeism. **Methods:** This multicenter prospective study will include 168 patients undergoing abdominal surgery. RedScar© utilizes smartphone-based automated infection risk assessments without clinician input. App-based detection will be compared with in-person evaluations. Sensitivity and specificity will be analyzed using receiver operating characteristic (ROC) analysis, while secondary objectives include assessing patient satisfaction and standardizing telematic follow-up. **Results:** This study aims to evaluate the efficacy of the RedScar© app, sensitivity, specificity in detecting SSIs. Satisfaction regarding comfort, cost-effectiveness, and absenteeism due to telematic detection and the monitoring of SSIs will be recorded too. **Conclusions:** This study seeks to validate RedScar© as a reliable and scalable tool for postoperative monitoring. By improving early SSI detection, it has the potential to enhance surgical recovery, reduce healthcare costs, and optimize resource utilization.

## 1. Introduction

Telematic follow-up in surgery has experienced significant growth, driven by technological advancements and social distancing measures stemming from the COVID-19 pandemic. Telemedicine offers numerous benefits, including remote consultations, preoperative evaluations, postoperative monitoring, and tele-mentoring, achieving high levels of user satisfaction [1,2]. The evolution of telemedicine has been marked by significant milestones in infection management, from early antiseptic techniques to modern digital monitoring tools. The ability to remotely assess surgical site infections (SSIs) represents a crucial advancement in preventing complications and optimizing healthcare resources.

Infections at the surgical site (SSI) represent the second most frequent type of healthcare-associated infection (HAI), comprising 19.6% of all HAIs across Europe [3]. Surgical site infections (SSIs) notably contribute to morbidity, leading to extended post-operative hospitalizations, the need for additional surgical interventions, higher mortality rates, and a negative impact on patients’ health-related quality of life (HRQoL) [4]. Recent epidemiological data indicate that SSI incidence varies between 0.5% and 10.1%, with higher rates in complex procedures. Additionally, 60% of SSIs are diagnosed post-discharge, emphasizing the importance of remote monitoring solutions [5,6].

In many cases, SSIs are diagnosed late, often after the patient has been discharged [6]. This delay exacerbates patient distress and significantly increases healthcare expenditures. In Europe, SSIs impose a considerable financial burden, with direct costs per infection estimated between EUR 10,443 and EUR 19,638 [7]. A comprehensive review highlights that these costs result from prolonged hospitalization, additional treatments, and outpatient visits. Furthermore, indirect costs—such as work absenteeism and long-term disability—pose further challenges to healthcare sustainability [8]. The early and effective detection of these infections post-discharge can prevent severe complications and optimize healthcare resources [9].

Several studies have explored the use of mobile applications for the telematic detection of SSIs. Some applications utilize thermal cameras to identify increases in temperature associated with infections [10], while others employ dressings monitored via mobile apps to detect or prevent infections at early stages [11]. However, these technologies are often complex and not widely applicable outside of research settings. The emergence of artificial intelligence (AI) and machine learning algorithms has opened new possibilities for SSI detection, with studies reporting predictive capabilities even before clinical symptoms appear [12]. Despite this progress, challenges related to cost, accessibility, and implementation hinder widespread adoption in clinical settings [12]

The RedScar© application v1.9 (Compilation 23) (University of the Balearic Islands, Palma, Spain) is designed to operate simply using a patient’s mobile phone and an internet connection, offering advantages over existing applications. Unlike previous studies that require the external evaluation of wound images [13].

The RedScar© app employs a comprehensive approach to assess surgical wound infections by integrating patient-reported data with automated image analysis. The user-friendly interface includes a structured questionnaire covering key wound-related factors such as pain severity (measured via a visual analog scale, VAS), redness, burning sensation, wound leakage, swelling, liquid secretion, and fever. Patients must complete all questions before proceeding to the next step, which involves uploading a wound photograph. Once submitted, the app initiates a multistep computerized image analysis process based on fuzzy sets and gray-scale mathematical morphology [14] which is then extended to color images [15]. By combining image analysis with questionnaire responses, RedScar© autonomously generates a diagnosis without requiring direct supervision from healthcare professionals. Developed in Java SE 11 (Oracle, Austin, TX, USA) using the Android Studio environment, RedScar© integrates advanced image-processing techniques through Python-based (Python 3.7, Python Software Foundation, Wilmington, DE, USA) repositories which localizes surgical staples and the wound in an image and reconstructs images.

RedScar© is fully automated, providing infection risk assessments without clinician input. Its independence from human intervention enhances scalability and cost-effectiveness in postoperative telemonitoring [16]. Utilizing real-time image processing and interactive questionnaires, RedScar© enables continuous patient engagement and rapid infection detection, potentially lowering rates of severe complications and hospital readmissions [17]. Previous studies have demonstrated that telemonitoring in surgery is feasible and can reduce the need for outpatient care while improving patient experiences [17,18,19].

The RedScar© Pilot Study [17]: Phase I/IIa evaluated the application for detecting and monitoring potentially infected surgical wounds. Among the 47 patients included, 41 successfully completed both remote and in-person follow-ups. The RedScar© system achieved a sensitivity of 100% for SSI detection, with a specificity of 83.13%. Patient satisfaction with telemonitoring was notably high: 97.6% considered it cost-effective, 90.47% believed it helped prevent absenteeism from work or school, and 80.9% found it a convenient option. While this pilot study confirmed the safety and feasibility of telematic follow-up using RedScar©, the low incidence of wound infections highlights the need for a multicenter trial to validate these findings and ensure standardized implementation across different healthcare centers. Our research aims to continue developing RedScar© as a user-friendly, reproducible tool to assist patients in managing surgical wounds at home, thus optimizing available resources.

## 2. Objectives

### 2.1. Primary Objectives

The primary objective will be to assess the sensitivity and specificity of the RedScar© application for detecting wound infection, comparing the app with in-person diagnosis. The ROC curve will be used to analyze the overall performance of the app and identify the optimal cut-off for the “Red Proportion” (maximizing sensitivity and specificity).

### 2.2. Secondary Objectives

The secondary objectives will be to verify the standardization of surgical wound follow-up using the RedScar© app across different centers and to assess the satisfaction level of subjects undergoing telemedicine-based follow-up using a validated telemedicine satisfaction questionnaire.

## 3. Methods

### 3.1. Study Design

This multicenter prospective study employs a paired cohort design with a non-randomized, single-blinded methodology, in which each participant acts as their own control. The paired design allows for more accurate within-subject comparisons, controlling for individual differences that could otherwise introduce bias. By having each participant act as their own control, we minimize confounding factors related to baseline characteristics, reducing the need for randomization. A randomized approach could introduce biases in patient expectations or compromise the practicality of using the application. The single-blind design, with outcome assessors blinded to the app’s recommendations, reduces assessment bias while maintaining practical feasibility. This design provides an optimal balance between minimizing confounding and ensuring the study’s practicality.

During wound assessments, investigators remain blinded to the application’s recommendations. None of the professionals participating in this study have access to patient information. This study will enroll adult patients undergoing abdominal surgery across multiple hospitals. This study involves the use of the RedScar© app (University of the Balearic Islands, Palma, Spain) on patients’ smartphones. Patients will complete a brief health questionnaire and upload a wound photograph to the app, which will provide recommendations based on the risk of infection detected. Simultaneously, an in-person wound assessment will be conducted by the investigator to compare results.

This study adheres to the 2015 guidelines established by the Standards for Reporting Diagnostic Accuracy Studies (STARD) initiative [20]. This ensures transparency and rigor in reporting diagnostic accuracy outcomes. The study’s design and protocol underwent a comprehensive review and received approval from the Research Ethics Committee of the Balearic Islands (CEI-IB) in 27 June 2023 under reference number IB 5087/22 PI. Informed consent will be obtained from all participants, guaranteeing patient confidentiality and data protection throughout this research. The study protocol was registered on ClinicalTrials.gov in 20 January 2025 under the identifier NCT06771726.

### 3.2. Sample Size and Recruitment

A minimum of 168 patients have been required to estimate the agreement between in-person and app-based evaluations for infection detection with a 95% confidence level and a precision of 0.12. The sample size has increased by 10% to account for potential patient loss, resulting in a total of 185 patients. The expected Kappa index is approximately 0.8, with an estimated infection prevalence of 15% (in-person) and 20% (app-based) based on our previous study. Sample size calculations were performed using Epidat 4.0 (Direccion Xeral de Innovación e Xestion da Saude Pública, Galicia, Spain).

Patients will be recruited by the investigators through hospital outpatient clinics and surgical departments.

### 3.3. Inclusion Criteria

The inclusion criteria require participants to have signed an informed consent form, be over 18 years of age, and have undergone either urgent or scheduled surgery performed via laparotomy or laparoscopy. Additionally, participants need access to a smartphone capable of downloading the app, with either the patient or a close family member being able to operate it effectively. Patients also must be able to attend follow-up consultations at the surgical outpatient clinic after discharge, one-week post-surgery, or earlier if the app flags a potential infection.

### 3.4. Exclusion Criteria

The exclusion criteria encompassed patients who lacked access to a smartphone or were unable to properly use the app, such as those unfamiliar with mobile devices or unable to comprehend the app’s functionality or questions. Patients who did not provide informed consent or who were unable to comply with the follow-up requirements were also excluded from this study.

### 3.5. Withdrawal Criteria

Patients may withdraw from this study at any time for any reason, and the reasons for withdrawal will be documented in the Case Report Form (CRF). These reasons may include the withdrawal of consent, the investigator’s discretion, or intercurrent illnesses that may compromise safety or interfere with this study. Once a patient has withdrawn, they will not be replaced or re-enrolled in this study.

### 3.6. Visit Schedule

The patient will be discharged after surgery when deemed appropriate by the attending physician.

They must download the RedScar© app before discharge so they can be taught how to use it correctly, and any questions regarding the app’s questionnaire will be addressed. Detailed explanations will be provided about changes in the wound, such as swelling, increased temperature, etc. As stated earlier, the inability to understand these conditions or use the app is a reason for exclusion.

**Figure 1:**
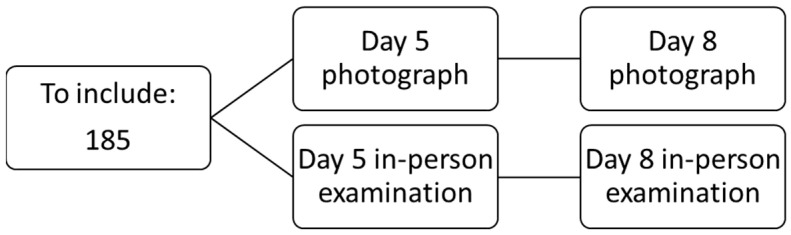
Flow chart.

The patient will attend their first follow-up at the surgical outpatient clinic on day 5 post-surgery to assess the wound and again on day 8 to remove staples and check the wound’s condition. Prior to the consultation, the patient will need to take a photograph of the wound using the app (on postoperative days 5 and 8) so the app’s results can be compared with the investigator’s in-person evaluation (Figure 1).

If a wound infection is suspected, the patient will need to visit the outpatient clinic or hospital emergency department (depending on the investigator’s guidelines) to be assessed by the main investigators or, if unavailable, by the attending nurse. They will confirm the presence or absence of wound infection, document it in the patient’s medical record, and perform the initial treatment. Additionally, the patient will be given a conventional follow-up appointment with the surgery clinic, as is performed for patients not included in this study.

At the end of the follow-up, patient satisfaction will be evaluated using the Telemedicine Satisfaction Questionnaire (Table 1) developed and validated by Yip et al. [2], which has been used in similar studies [17,18].

## 4. Data Analysis

### 4.1. Study Variables

The personal data collected includes ID number, hospital record number, name, surname, and phone number. Study variables include sex, age, medical history, ASA classification, pathology, date and type of surgery (open/laparoscopic), type of wound (McBurney incision/trocar/midline laparotomy/other), date of discharge, days of hospitalization, and satisfaction level.

### 4.2. Statistical Analysis

Data will be entered into data entry programs that ensure the integrity of the information. For this, programs that comply with data validation guidelines and software published by regulatory agencies will be used. The analysis plan will be prepared before the database is closed.

Quantitative variables will be analyzed descriptively using means and standard deviations or medians and percentiles, accompanied by a bilateral 95% confidence interval and range (minimum and maximum) or P50 [P25–P75]. Qualitative variables will be summarized in a table with both absolute and relative frequencies. Statistical significance will be assessed at *p* < 0.05. Data analysis will be conducted using IBM^®^ SPSS^®^ Statistics 19 software.

## 5. Data Management

### 5.1. Patient Registry

To enable patient access to the application, a selection and inclusion registry will be created, establishing a file where all the data of the patients included in this study will be recorded. Both paper and electronic formats will be used to retain the informed consent, the approved study protocol, and copies of the data collection forms. The reason for excluding a patient from this study will be specified for each case.

**Figure 2:**
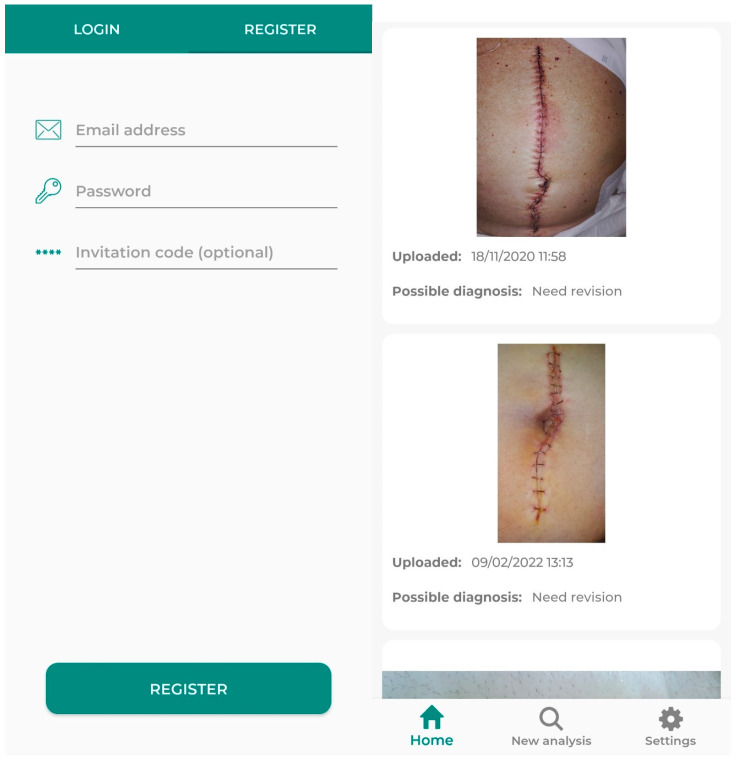
Interface that the user finds when registering (**left**), and when finishing registration and accessing the patient area (**right**).

A randomly generated code will be assigned to the selected patients, created automatically by the application. This function will be found in the healthcare provider’s section of the application. Among other actions, it can generate codes for patient registration, view images uploaded by the patient (with the appropriate permission), and assess the patient’s progress. In this way, only the investigator will know the correspondence between the patient and the code, as during the code generation process, the patient’s name or any identifying element will not be required. The patient will then use this code to register in the application (Figure 2). To complete the registration, the patient will need to enter their own credentials to avoid having to periodically input the code. The registration process ends by providing access to the patient area (Figure 2), where the patient can evaluate the automatic infection analysis integrated into the app.

### 5.2. Image Storage

**Figure 3:**
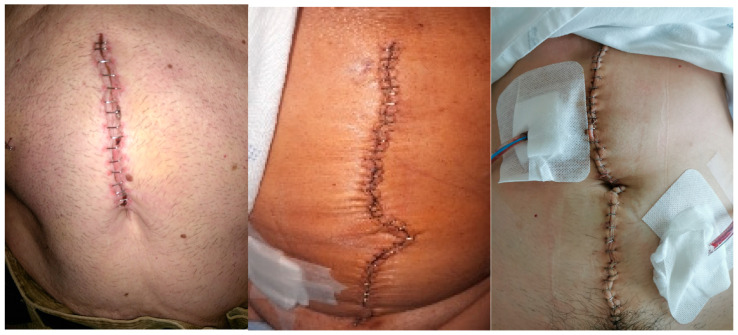
Examples of images that are expected to be included in this study, taken by patients in the preliminary phase of this study (see the RedScar© Pilot Study [15]).

The images that we intend to collect in this phase of the study are similar to those shown in Figure 3. When a patient initiates a new automatic analysis, the attached image will be transmitted to the application’s server for processing using the infection detection method. Once the analysis is completed, since the patient has been included in this study and has signed the document authorizing subsequent use for scientific purposes, the image is stored in a database specifically created to hold the images included in this study.

### 5.3. Server Composition

The application is supported by two servers. On one hand, the computing server, which manages any interaction with the application, such as validating credentials, creating new users, etc., operates via a secure and encrypted connection. Additionally, it also performs the automatic image analysis. This service is located at the University of the Balearic Islands (UIB), and all connections are secured via the HTTPS protocol.

On the other hand, the server that hosts the entire database is located on a virtual machine within Google’s Firebase service. In this database, all information is encrypted and only accessible to the project’s responsible researchers. The connection with the applications on this service is also secure through the HTTPS protocol, and no database reading is allowed unless it is from the processing server located at UIB.

Among the services provided by both servers is automatic notification to researchers via email when a patient uploads an image with research permission, as well as user password recovery via email.

### 5.4. Structure of Personal Information

Patient information will be stored in a JSON format, utilizing a key-value system. Researchers will also have accounts within the same system, each with a unique identifier that links them to the patients they are associated with. Furthermore, multiple researchers may be linked to a single patient.

As for the account parameters, the ENABLED_ACCOUNT parameter allows remote control of the user’s account status in case they cease collaboration. Regarding the images, the same principle applies as with the researchers: it is a list of image-type objects, and there will be as many as the patient has chosen to upload. The two permissions mentioned previously will also apply.

Although it has been mentioned multiple times, it is worth reiterating that all key-value information in the JSON file is encrypted.

## 6. Ethical Considerations

### 6.1. Security and Confidentiality Measures

The contents of the data collection form and the confidentiality of each patient’s information will be fully upheld.

Appropriate procedures will be followed to ensure compliance with Spanish Organic Law 3/2018, dated 5 December, regarding the Protection of Personal Data and the safeguarding of digital rights. Documents generated throughout this study will be secured against unauthorized access by individuals outside the research team and will remain strictly confidential. They will not be shared with third parties except as outlined in the previous section. The investigator will inform participants that the data collected in this study will be stored and analyzed electronically, in accordance with Spanish regulations on the management of computerized data.

The confidentiality of patients will be maintained at all times, and only the investigator will know the patient’s personal data and location, in case authorities require them according to current legislation procedures.

With the new General Data Protection Regulation (GDPR) [21], the obligation to declare data files is removed, though not the obligation to maintain a record of them. As the Regulation itself establishes, “Each data controller and, where applicable, their representative, shall maintain a record of processing activities under their responsibility.” Thus, the formal obligation to report files is eliminated, but they must still be clearly identified.

The information disseminated and obtained from the implementation of this study will be considered confidential and always be treated as such. Study participants will be identified only by their study code. If the study results are published, the identity of the volunteers will not be revealed.

Volunteers may exercise their right to access, rectify, cancel, and oppose their personal data at any time.

### 6.2. Informed Consent

Before starting this study, all participants will be informed and will give their consent in writing, with a duplicate copy. Both the patient information sheet and the informed consent form are included in the annex. Participants will be given a copy of this information sheet to keep. Only patients who sign the consent form after being informed will be included.

## Figures and Tables

**Table 1:** Satisfaction questionnaire [2].

Satisfaction Questionnaire	Answer
1. The information received before participating in this study was adequate.	Yes/No
2. I do not have difficulties with the questionnaire used within the application.	Yes/No
3. I do not have difficulties when taking the photo and uploading it to the mobile application.	Yes/No
4. I have spent less time with telematic follow-up than with personal revision.	Yes/No
5. I think that telematic follow-up is cheaper than the personal revision.	Yes/No
6. I think that telematic review could prevent absenteeism from work/school.	Yes/No
7. Telematic follow-up seems more comfortable to me than follow-up in consultations.	Yes/No
8. I think that telematic monitoring could detect a bad evolution earlier.	Yes/No
9. I think the healthcare provided via telemedicine is consistent.	Yes/No
10. The care received seems adequate to me.	Yes/No
11. Overall, I am satisfied with the quality of service being provided via telemedicine.	Yes/No
12. I would prefer telematic follow-up to face-to-face consultations.	Yes/No
13. I will use telemedicine services again.	Yes/No
14. Following the instructions of the application, have you checked your wound at your health center?	Yes/No, Why?
15. Although the application had ruled out the infection of the wound, have you preferred to check it in person?	Yes/No, Why?
16. Despite using the application, have you consulted any other source (Google-type web search, opinion of close relatives)?	Yes/No, Why?
17. How do you think we can improve the RedScar© application? Leave your comment.	

## Data Availability

Data are available through a virtual repository of surgical wound photographs. The images are anonymized, making it impossible to recognize any patient, in accordance with CEE regulations. This repository can be accessed through the website redscar.uib.es (accessed on 10 December 2024) [22].
